# μ-1,6,7,12-Tetra­aza­perylene-κ^4^ *N* ^1^,*N* ^12^:*N* ^6^,*N* ^7^-bis­[chlorido­(η^6^-*p*-cymene)ruthenium(II)] bis­(hexa­fluorido­phosphate) acetone disolvate

**DOI:** 10.1107/S160053681400035X

**Published:** 2014-01-15

**Authors:** Thomas Brietzke, Daniel Kässler, Alexandra Kelling, Uwe Schilde, Hans-Jürgen Holdt

**Affiliations:** a Universität Potsdam, Institut für Chemie, Anorganische Chemie, Karl-Liebknecht-Strasse 24-25, D-14476 Potsdam, Germany

## Abstract

In the title compound, [Ru_2_(C_10_H_14_)_2_Cl_2_(C_16_H_8_N_4_)](PF_6_)_2_·2C_3_H_6_O, the binuclear Ru^II^ complex dication, [{RuCl(η^6^-cym)}_2_(μ-tape)]^2+^, built up by a planar 1,6,7,12-tetra­aza­perylene (tape) bridge, two η^6^-bound cymene (cym) ligands and two chloride ligands, includes an inversion center. The Ru^II^ atom shows the typical piano-stool motif for arene coordination. The counter-charge is provided by a hexa­fluorido­phosphate anion and the asymmetric unit is completed by an acetone mol­ecule of crystallization. The components of the structure are connected into a three-dimensional architecture by C—H⋯O/F/Cl inter­actions.

## Related literature   

For related Ru^II^–arene complexes, see: Bennett & Smith (1974 ▶); Robertson *et al.* (1980 ▶); Govindaswamy *et al.* (2007 ▶); Betanzos-Lara *et al.* (2012 ▶). For tetra­aza­perylene-bridged Ru^II^ complexes, see: Brietzke, Mickler, Kelling & Holdt (2012 ▶); Brietzke, Mickler, Kelling, Schilde *et al.* (2012 ▶).
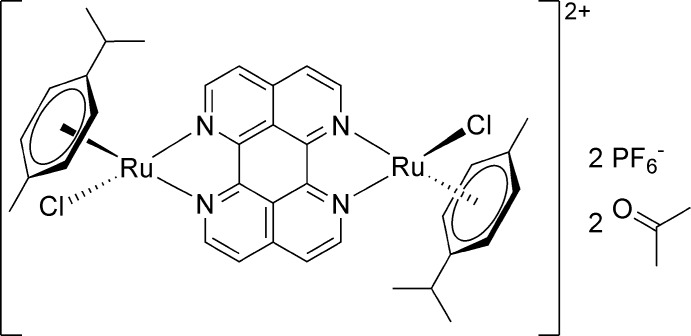


## Experimental   

### 

#### Crystal data  

[Ru_2_(C_10_H_14_)_2_Cl_2_(C_16_H_8_N_4_)](PF_6_)_2_·2C_3_H_6_O
*M* *_r_* = 1203.82
Triclinic, 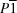

*a* = 8.6289 (5) Å
*b* = 11.9346 (7) Å
*c* = 12.7785 (7) Å
α = 66.099 (4)°
β = 83.536 (4)°
γ = 77.572 (4)°
*V* = 1174.45 (12) Å^3^
*Z* = 1
Mo *K*α radiation
μ = 0.91 mm^−1^
*T* = 293 K
1.50 × 0.62 × 0.17 mm

#### Data collection  

Stoe IPDS-2 diffractometer
Absorption correction: integration (*X-RED*; Stoe & Cie, 2011 ▶) *T*_min_ = 0.614, *T*_max_ = 0.860
15353 measured reflections
4133 independent reflections
3971 reflections with *I* > 2σ(*I*)
*R*_int_ = 0.047

#### Refinement  

*R*[*F* ^2^ > 2σ(*F* ^2^)] = 0.030
*wR*(*F* ^2^) = 0.080
*S* = 1.01
4133 reflections
302 parameters
H-atom parameters constrained
Δρ_max_ = 1.17 e Å^−3^
Δρ_min_ = −0.67 e Å^−3^

### 

Data collection: *X-AREA* (Stoe & Cie, 2011 ▶); cell refinement: *X-AREA*; data reduction: *X-RED* (Stoe & Cie, 2011 ▶); program(s) used to solve structure: *SHELXS97* (Sheldrick, 2008 ▶); program(s) used to refine structure: *SHELXL2013* (Sheldrick, 2008 ▶); molecular graphics: *DIAMOND* (Brandenburg, 2012 ▶) and *ORTEP-3 for Windows* (Farrugia, 2012 ▶); software used to prepare material for publication: *SHELXL2013*.

## Supplementary Material

Crystal structure: contains datablock(s) global, I. DOI: 10.1107/S160053681400035X/tk5277sup1.cif

Structure factors: contains datablock(s) I. DOI: 10.1107/S160053681400035X/tk5277Isup2.hkl

CCDC reference:

Additional supporting information:  crystallographic information; 3D view; checkCIF report

## Figures and Tables

**Table 1:** Selected bond lengths (Å)

C9—Ru1	2.214 (2)
C10—Ru1	2.188 (2)
C11—Ru1	2.204 (3)
C12—Ru1	2.212 (3)
C13—Ru1	2.185 (3)
C14—Ru1	2.200 (3)
Cl1—Ru1	2.3844 (7)
N1—Ru1	2.105 (2)
N2—Ru1	2.105 (2)

**Table 2:** Hydrogen-bond geometry (Å, °)

*D*—H⋯*A*	*D*—H	H⋯*A*	*D*⋯*A*	*D*—H⋯*A*
C1—H1⋯O1	0.93	2.39	3.194 (4)	145
C3—H3⋯Cl1^i^	0.93	2.84	3.637 (3)	145
C3—H3⋯F6^ii^	0.93	2.58	3.205 (4)	125
C4—H4⋯F6^ii^	0.93	2.60	3.223 (4)	125
C19—H19*B*⋯F4^iii^	0.96	2.46	3.321 (5)	149

Symmetry codes: (i) 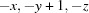; (ii) 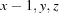; (iii) 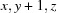.

**Fig. 1.**
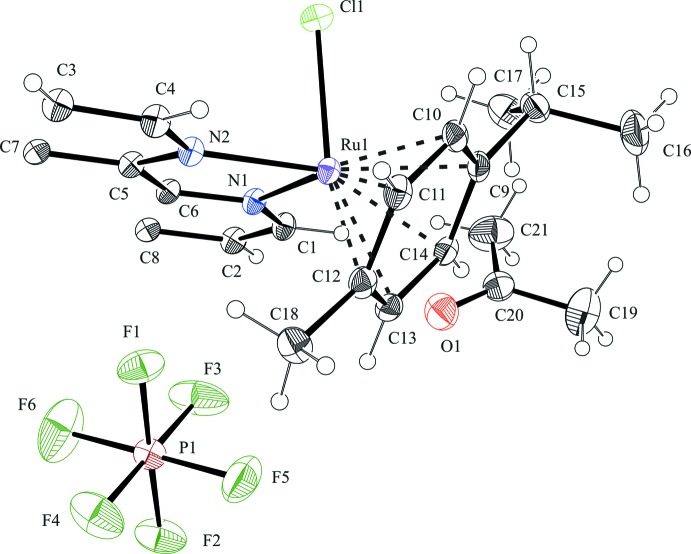
ORTEP drawing of asymmetric unit with the atomic numbering scheme and 30% probability displacement ellipsoids for non-hydrogen atoms.

**Fig. 2.**
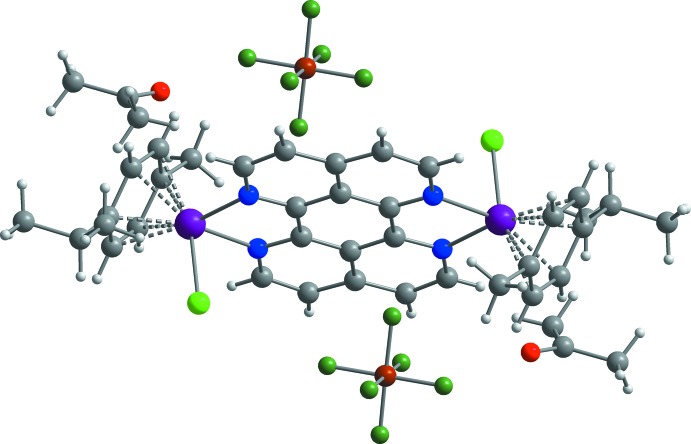
The molecular structure of the title compound.

**Fig. 3.**
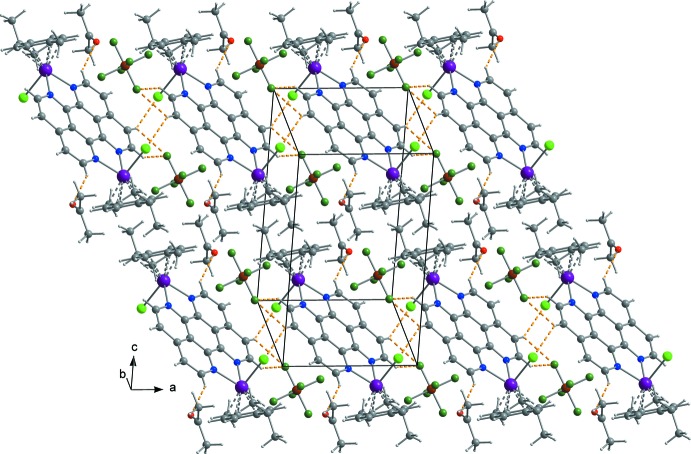
A packing diagram of the title compound is displayed approximately along the b axis. Hydrogen bonds are shown as orange dashed lines. One C—H···F hydrogen bond running along b was omitted for clarity.

## supplementary crystallographic information

### 2.1. Synthesis and crystallization

[RuCl_2_(*η*^6^-cym)]_2_ was prepared as described in literature (Bennett & Smith, 1974). [{RuCl(*η*^6^-cym)}_2_(µ-tape)](PF_6_)_2_ was prepared in the same way as described in literature for [RuCl(*η*^6^-cym)phen]PF_6_ (Robertson *et al.*, 1980) using only one eqivalent tape instead of an excess of phenanthroline (phen). Crystals suitable for X-ray structure analysis were obtained by vapor diffusion of di­ethyl ether into a saturated acetone solution of [{RuCl(*η*^6^-cym)}_2_(µ-tape)](PF_6_)_2_. Therefore, the solution was placed into a flask, connected with another di­ethyl­ether containing flask and a slight vacuum was applied. Dark crystals began to form at ambient temperature within one night.

### 2.2. Refinement

All hydrogen atoms were calculated in their expected positions and refined as riding atoms with *U*_iso_(H)=1.2*U*_eq_(C) with the exception of methyl hydrogen atoms, which were refined with *U*_iso_(H)=1.5*U*_eq_(C). The maximum residual electron density peak of 1.17 eÅ^-3^ was located 1.45 Å from the Ru atom.

### 3. Results and discussion

1,6,7,12-Tetra­aza­perylene (tape) is a *D*_2h_-symmetric bis­(α,α'-di­imine)-type bridging ligand containing an extended π-heteroaromatic system. As for 2,2'-bi­pyrimidine (bpym), tape can be used to construct a series of dinuclear metal complexes. These dinuclear Ru^II^ complexes of tape display intense low-energy dπ(Ru)→π* (tape) MLCT absorption bands. Separated metal oxidations for the bimetallic tape complexes and the inter­valence-charge-transfer (IVCT) transition, measured for [{Ru(L–N_4_Me_2_)}_2_(µ-tape)]^5+^, where L–N_4_Me_2_ is *N*,*N*'-di­methyl-2,11-di­aza­[3.3](2,6)-pyridino­phane, at 2472 nm indicate a high degree of electronic inter­action between the two ruthenium centres, mediated through the tape bridging ligand (Brietzke, Mickler, Kelling & Holdt, 2012; Brietzke, Mickler, Kelling, Schilde *et al.*, 2012). Here, we report the crystal structure of µ_2_-1,6,7,12-tetra­aza­perylene-bis­[chlorido-(*η*^6^-*p*-cymene)-ruthenium(II)] bis­(hexa­fluoro­phosphate) acetone solvate, Fig. 1 & Table 1. The *p*-cymene (cym) ligands are coordinated to ruthenium in the typical piano-stool like mode. Comparison of the published [{RuCl(*η*^6^-cym)}_2_(µ-bpym)](PF_6_)_2_ structure (Govindaswamy *et al.*, 2007) and [{RuCl(*η*^6^-cym)}_2_(µ-tape)](PF_6_)_2_ show slightly shorter Ru—N distances for the tape complex (Ru—N1, Ru—N2 = 2.117 (3) Å, 2.122 (3) Å and 2.105 (2) Å, 2.105 (2) Å for the bpym and tape complexes, respectively). These shorter bonds should be mainly lead back to the slightly higher π-acceptor strength of tape. Furthermore, only very small differences in the distance between ruthenium(II) and the *p*-cymene centroid (1.6886 (3) Å and 1.6847 (2) Å for bpym and tape complex, respectively) and excactly the same Ru—Cl bond length (2.384 (1) Å, 2.384 (7) Å) were observed. The Ru—Ru distance in [{RuCl(*η*^6^-cym)}_2_(µ-tape)](PF_6_)_2_ is 8.0715 (5) Å, and therefore 0.088 Å longer than in [{Ru(L—N_4_Me_2_)}_2_(µ-tape)](PF_6_)_4_ (Brietzke, Mickler, Kelling, Schilde *et al.*, 2012), what is affected by longer Ru—N bonds in the arene complex. However, the Ru—N bond lengths (were N is part of a bpy type ligand) of [{RuCl(*η*^6^-cym)}_2_(µ-tape)](PF_6_)_2_ are closely related (Ru—N(bpy type) 2.121-2.080 Å) with earlier reported ruthenium arene complexes (Betanzos-Lara *et al.*, 2012; Govindaswamy *et al.*, 2007). The three-dimensional structure of the title compound is build from staggered layers, spaced by *p*-cymene moieties, hexa­fluoro­phosphate anions and the acetone solvent molecules. In the crystal structure the tape moieties inter­act *via* C3 with chlorine and hexa­fluoro­phosphate (F6), which is also linked to the adjacent C4, forming bifurcurated non classical hydrogen bonds, Table 2. Further weak hydrogen bonds can be found between tape (C1) and acetone (O1), with the result that acetone build a bridge *via* C19 to hexa­fluoro­phosphate (F4), Fig. 3.

### Crystal data

**Table:**

[Ru_2_(C_10_H_14_)_2_Cl_2_(C_16_H_8_N_4_)](PF_6_)_2_·2C_3_H_6_O	*Z* = 1
*M**_r_* = 1203.82	*F*(000) = 604
Triclinic, *P*1	*D*_x_ = 1.702 Mg m^−^^3^
Hall symbol: -P 1	Mo *K*α radiation, λ = 0.71073 Å
*a* = 8.6289 (5) Å	Cell parameters from 20121 reflections
*b* = 11.9346 (7) Å	θ = 1.7–29.8°
*c* = 12.7785 (7) Å	µ = 0.91 mm^−^^1^
α = 66.099 (4)°	*T* = 293 K
β = 83.536 (4)°	Needle, black
γ = 77.572 (4)°	1.50 × 0.62 × 0.17 mm
*V* = 1174.45 (12) Å^3^	

### Data collection

**Table:**

Stoe IPDS-2 diffractometer	4133 independent reflections
Radiation source: sealed X-ray tube	3971 reflections with *I* > 2σ(*I*)
Detector resolution: 6.67 pixels mm^-1^	*R*_int_ = 0.047
ω scan	θ_max_ = 25.0°, θ_min_ = 1.7°
Absorption correction: integration (*X-RED*; Stoe & Cie, 2011)	*h* = −9→10
*T*_min_ = 0.614, *T*_max_ = 0.860	*k* = −14→14
15353 measured reflections	*l* = −15→15

### Refinement

**Table:**

Refinement on *F*^2^	Secondary atom site location: difference Fourier map
Least-squares matrix: full	Hydrogen site location: inferred from neighbouring sites
*R*[*F*^2^ > 2σ(*F*^2^)] = 0.030	H-atom parameters constrained
*wR*(*F*^2^) = 0.080	*w* = 1/[σ^2^(*F*_o_^2^) + (0.045*P*)^2^ + 1.286*P*] where *P* = (*F*_o_^2^ + 2*F*_c_^2^)/3
*S* = 1.01	(Δ/σ)_max_ = 0.003
4133 reflections	Δρ_max_ = 1.17 e Å^−^^3^
302 parameters	Δρ_min_ = −0.67 e Å^−^^3^
0 restraints	Extinction correction: *SHELXL2013* (Sheldrick, 2008), Fc^*^=kFc[1+0.001xFc^2^λ^3^/sin(2θ)]^-1/4^
Primary atom site location: structure-invariant direct methods	Extinction coefficient: 0.0088 (8)

### Special details

**Table:**

Geometry. All e.s.d.'s (except the e.s.d. in the dihedral angle between two l.s. planes) are estimated using the full covariance matrix. The cell e.s.d.'s are taken into account individually in the estimation of e.s.d.'s in distances, angles and torsion angles; correlations between e.s.d.'s in cell parameters are only used when they are defined by crystal symmetry. An approximate (isotropic) treatment of cell e.s.d.'s is used for estimating e.s.d.'s involving l.s. planes.
Refinement. Refinement of *F*^2^ against ALL reflections. The weighted *R*-factor *wR* and goodness of fit *S* are based on *F*^2^, conventional *R*-factors *R* are based on *F*, with *F* set to zero for negative *F*^2^. The threshold expression of *F*^2^ > 2σ(*F*^2^) is used only for calculating *R*-factors(gt) *etc*. and is not relevant to the choice of reflections for refinement. *R*-factors based on *F*^2^ are statistically about twice as large as those based on *F*, and *R*-factors based on all data will be even larger.

### Fractional atomic coordinates and isotropic or equivalent isotropic displacement parameters (Å^2^)

**Table:**

	*x*	*y*	*z*	*U*_iso_*/*U*_eq_	
C1	0.5372 (3)	0.7198 (2)	0.1198 (2)	0.0278 (5)	
H1	0.5127	0.7695	0.1619	0.033*	
C2	0.6772 (3)	0.7223 (2)	0.0576 (2)	0.0282 (5)	
H2	0.7457	0.7720	0.0592	0.034*	
C3	0.1439 (3)	0.3571 (2)	0.0791 (2)	0.0288 (5)	
H3	0.0679	0.3099	0.0853	0.035*	
C4	0.1221 (3)	0.4332 (2)	0.1380 (2)	0.0293 (5)	
H4	0.0302	0.4364	0.1831	0.035*	
C5	0.3591 (3)	0.5000 (2)	0.0674 (2)	0.0237 (5)	
C6	0.4691 (3)	0.5778 (2)	0.0619 (2)	0.0231 (5)	
C7	0.3911 (3)	0.4242 (2)	0.0050 (2)	0.0231 (5)	
C8	0.7183 (3)	0.6500 (2)	−0.0090 (2)	0.0250 (5)	
C9	0.1106 (3)	0.7601 (3)	0.2988 (2)	0.0302 (6)	
C10	−0.0022 (3)	0.6859 (3)	0.3042 (2)	0.0313 (6)	
H10	−0.1038	0.7252	0.2778	0.038*	
C11	0.0364 (3)	0.5555 (3)	0.3479 (2)	0.0312 (6)	
H11	−0.0400	0.5097	0.3515	0.037*	
C12	0.1928 (3)	0.4922 (2)	0.3873 (2)	0.0314 (6)	
C13	0.3041 (3)	0.5644 (3)	0.3818 (2)	0.0307 (6)	
H13	0.4067	0.5251	0.4062	0.037*	
C14	0.2629 (3)	0.6972 (2)	0.3395 (2)	0.0292 (5)	
H14	0.3380	0.7428	0.3389	0.035*	
C15	0.0633 (4)	0.9007 (3)	0.2507 (3)	0.0383 (7)	
H15	−0.0254	0.9237	0.1999	0.046*	
C16	0.0016 (5)	0.9403 (3)	0.3501 (3)	0.0629 (11)	
H16A	0.0839	0.9136	0.4044	0.094*	
H16B	−0.0883	0.9027	0.3868	0.094*	
H16C	−0.0294	1.0295	0.3213	0.094*	
C17	0.1938 (4)	0.9683 (3)	0.1810 (3)	0.0527 (8)	
H17A	0.2254	0.9444	0.1174	0.079*	
H17B	0.2834	0.9467	0.2283	0.079*	
H17C	0.1555	1.0567	0.1532	0.079*	
C18	0.2359 (4)	0.3522 (3)	0.4299 (3)	0.0396 (7)	
H18A	0.1643	0.3221	0.3997	0.059*	
H18B	0.2284	0.3167	0.5120	0.059*	
H18C	0.3426	0.3287	0.4051	0.059*	
C19	0.5166 (6)	0.9785 (4)	0.3738 (4)	0.0748 (12)	
H19A	0.4216	1.0407	0.3534	0.112*	
H19B	0.5910	1.0067	0.4036	0.112*	
H19C	0.4914	0.9021	0.4310	0.112*	
C20	0.5881 (4)	0.9566 (3)	0.2701 (3)	0.0446 (7)	
C21	0.6172 (6)	1.0667 (5)	0.1694 (4)	0.0792 (13)	
H21A	0.6894	1.1064	0.1881	0.119*	
H21B	0.5188	1.1241	0.1458	0.119*	
H21C	0.6625	1.0419	0.1082	0.119*	
Cl1	0.09547 (8)	0.78153 (6)	0.03483 (5)	0.03517 (17)	
F1	0.5627 (2)	0.3410 (2)	0.26787 (17)	0.0589 (5)	
F2	0.8898 (2)	0.3000 (2)	0.3836 (2)	0.0675 (6)	
F3	0.7334 (4)	0.4617 (2)	0.2626 (3)	0.0938 (10)	
F4	0.7241 (3)	0.1753 (2)	0.3893 (3)	0.0811 (8)	
F5	0.6323 (3)	0.3400 (3)	0.4315 (2)	0.0837 (8)	
F6	0.8209 (3)	0.2954 (4)	0.2213 (3)	0.1058 (11)	
N1	0.4310 (2)	0.64737 (19)	0.12313 (17)	0.0247 (4)	
N2	0.2288 (2)	0.50461 (19)	0.13364 (17)	0.0253 (4)	
O1	0.6209 (3)	0.8519 (2)	0.2737 (2)	0.0611 (7)	
P1	0.72700 (9)	0.31873 (7)	0.32587 (7)	0.03586 (18)	
Ru1	0.20978 (2)	0.63314 (2)	0.21078 (2)	0.02344 (10)	

### Atomic displacement parameters (Å^2^)

**Table:**

	*U*^11^	*U*^22^	*U*^33^	*U*^12^	*U*^13^	*U*^23^
C1	0.0287 (13)	0.0305 (12)	0.0306 (12)	−0.0114 (11)	0.0015 (11)	−0.0163 (10)
C2	0.0275 (13)	0.0300 (12)	0.0326 (13)	−0.0140 (11)	0.0003 (11)	−0.0139 (11)
C3	0.0250 (12)	0.0325 (13)	0.0336 (13)	−0.0145 (11)	0.0032 (11)	−0.0141 (11)
C4	0.0233 (12)	0.0356 (13)	0.0336 (13)	−0.0125 (11)	0.0061 (11)	−0.0166 (11)
C5	0.0224 (11)	0.0265 (11)	0.0231 (11)	−0.0090 (10)	0.0017 (9)	−0.0092 (9)
C6	0.0232 (12)	0.0268 (11)	0.0217 (11)	−0.0091 (10)	0.0007 (9)	−0.0103 (9)
C7	0.0225 (12)	0.0254 (11)	0.0231 (11)	−0.0091 (10)	−0.0010 (10)	−0.0088 (9)
C8	0.0241 (12)	0.0267 (12)	0.0258 (12)	−0.0098 (10)	−0.0007 (10)	−0.0094 (10)
C9	0.0299 (13)	0.0352 (14)	0.0296 (13)	−0.0034 (11)	0.0049 (11)	−0.0197 (11)
C10	0.0230 (12)	0.0410 (14)	0.0325 (13)	−0.0035 (11)	0.0058 (11)	−0.0199 (12)
C11	0.0277 (13)	0.0392 (14)	0.0308 (13)	−0.0126 (11)	0.0100 (11)	−0.0176 (11)
C12	0.0349 (14)	0.0333 (13)	0.0241 (12)	−0.0078 (12)	0.0068 (11)	−0.0107 (10)
C13	0.0303 (13)	0.0366 (14)	0.0239 (12)	−0.0040 (11)	0.0004 (10)	−0.0120 (11)
C14	0.0306 (13)	0.0370 (14)	0.0268 (12)	−0.0086 (11)	0.0011 (10)	−0.0186 (11)
C15	0.0356 (15)	0.0341 (14)	0.0459 (16)	−0.0002 (12)	−0.0046 (13)	−0.0186 (13)
C16	0.079 (3)	0.0439 (18)	0.068 (2)	0.0035 (18)	0.009 (2)	−0.0350 (18)
C17	0.0503 (19)	0.0348 (16)	0.067 (2)	−0.0084 (14)	−0.0035 (17)	−0.0128 (15)
C18	0.0425 (16)	0.0319 (14)	0.0383 (15)	−0.0088 (13)	0.0075 (13)	−0.0090 (12)
C19	0.088 (3)	0.084 (3)	0.073 (3)	−0.039 (3)	0.021 (2)	−0.046 (2)
C20	0.0364 (16)	0.0520 (19)	0.0522 (18)	−0.0107 (14)	−0.0066 (14)	−0.0248 (15)
C21	0.076 (3)	0.085 (3)	0.057 (2)	−0.024 (3)	−0.001 (2)	−0.005 (2)
Cl1	0.0354 (3)	0.0383 (3)	0.0310 (3)	−0.0088 (3)	−0.0050 (3)	−0.0108 (3)
F1	0.0433 (11)	0.0809 (14)	0.0505 (11)	−0.0207 (10)	−0.0098 (9)	−0.0170 (10)
F2	0.0412 (11)	0.0592 (12)	0.0888 (16)	−0.0176 (10)	−0.0221 (11)	−0.0062 (11)
F3	0.104 (2)	0.0417 (12)	0.116 (2)	−0.0260 (13)	−0.0552 (18)	0.0085 (13)
F4	0.0662 (15)	0.0428 (11)	0.124 (2)	−0.0188 (11)	−0.0134 (15)	−0.0148 (13)
F5	0.0653 (15)	0.138 (2)	0.0661 (14)	−0.0051 (15)	−0.0010 (12)	−0.0656 (16)
F6	0.0733 (17)	0.181 (3)	0.103 (2)	−0.053 (2)	0.0486 (16)	−0.094 (2)
N1	0.0239 (10)	0.0284 (10)	0.0254 (10)	−0.0091 (9)	0.0016 (8)	−0.0128 (8)
N2	0.0240 (10)	0.0289 (10)	0.0265 (10)	−0.0100 (9)	0.0037 (9)	−0.0128 (9)
O1	0.0586 (15)	0.0611 (16)	0.0792 (18)	−0.0093 (13)	−0.0124 (14)	−0.0416 (14)
P1	0.0298 (4)	0.0372 (4)	0.0405 (4)	−0.0110 (3)	0.0007 (3)	−0.0131 (3)
Ru1	0.02053 (13)	0.02803 (14)	0.02507 (14)	−0.00703 (9)	0.00287 (9)	−0.01342 (9)

### Geometric parameters (Å, º)

**Table:**

C1—C2	1.368 (4)	C13—H13	0.9300
C1—N1	1.376 (3)	C14—Ru1	2.200 (3)
C1—H1	0.9300	C14—H14	0.9300
C2—C8	1.411 (4)	C15—C17	1.515 (5)
C2—H2	0.9300	C15—C16	1.531 (4)
C3—C4	1.369 (4)	C15—H15	0.9800
C3—C8^i^	1.415 (4)	C16—H16A	0.9600
C3—H3	0.9300	C16—H16B	0.9600
C4—N2	1.366 (3)	C16—H16C	0.9600
C4—H4	0.9300	C17—H17A	0.9600
C5—N2	1.335 (3)	C17—H17B	0.9600
C5—C7	1.399 (3)	C17—H17C	0.9600
C5—C6	1.442 (3)	C18—H18A	0.9600
C6—N1	1.327 (3)	C18—H18B	0.9600
C6—C7^i^	1.400 (3)	C18—H18C	0.9600
C7—C6^i^	1.400 (3)	C19—C20	1.493 (5)
C7—C8^i^	1.410 (3)	C19—H19A	0.9600
C8—C7^i^	1.410 (3)	C19—H19B	0.9600
C8—C3^i^	1.415 (4)	C19—H19C	0.9600
C9—C14	1.405 (4)	C20—O1	1.203 (4)
C9—C10	1.430 (4)	C20—C21	1.465 (5)
C9—C15	1.512 (4)	C21—H21A	0.9600
C9—Ru1	2.214 (2)	C21—H21B	0.9600
C10—C11	1.399 (4)	C21—H21C	0.9600
C10—Ru1	2.188 (2)	Cl1—Ru1	2.3844 (7)
C10—H10	0.9300	F1—P1	1.595 (2)
C11—C12	1.432 (4)	F2—P1	1.592 (2)
C11—Ru1	2.204 (3)	F3—P1	1.575 (2)
C11—H11	0.9300	F4—P1	1.574 (2)
C12—C13	1.401 (4)	F5—P1	1.579 (2)
C12—C18	1.506 (4)	F6—P1	1.574 (3)
C12—Ru1	2.212 (3)	N1—Ru1	2.105 (2)
C13—C14	1.427 (4)	N2—Ru1	2.105 (2)
C13—Ru1	2.185 (3)		
			
C2—C1—N1	123.2 (2)	C12—C18—H18A	109.5
C2—C1—H1	118.4	C12—C18—H18B	109.5
N1—C1—H1	118.4	H18A—C18—H18B	109.5
C1—C2—C8	120.4 (2)	C12—C18—H18C	109.5
C1—C2—H2	119.8	H18A—C18—H18C	109.5
C8—C2—H2	119.8	H18B—C18—H18C	109.5
C4—C3—C8^i^	120.2 (2)	C20—C19—H19A	109.5
C4—C3—H3	119.9	C20—C19—H19B	109.5
C8^i^—C3—H3	119.9	H19A—C19—H19B	109.5
N2—C4—C3	123.2 (2)	C20—C19—H19C	109.5
N2—C4—H4	118.4	H19A—C19—H19C	109.5
C3—C4—H4	118.4	H19B—C19—H19C	109.5
N2—C5—C7	122.9 (2)	O1—C20—C21	123.9 (4)
N2—C5—C6	117.0 (2)	O1—C20—C19	119.5 (4)
C7—C5—C6	120.2 (2)	C21—C20—C19	116.6 (4)
N1—C6—C7^i^	123.5 (2)	C20—C21—H21A	109.5
N1—C6—C5	116.9 (2)	C20—C21—H21B	109.5
C7^i^—C6—C5	119.7 (2)	H21A—C21—H21B	109.5
C6^i^—C7—C5	120.2 (2)	C20—C21—H21C	109.5
C6^i^—C7—C8^i^	119.9 (2)	H21A—C21—H21C	109.5
C5—C7—C8^i^	119.9 (2)	H21B—C21—H21C	109.5
C2—C8—C7^i^	116.0 (2)	C6—N1—C1	117.0 (2)
C2—C8—C3^i^	127.8 (2)	C6—N1—Ru1	114.24 (17)
C7^i^—C8—C3^i^	116.2 (2)	C1—N1—Ru1	128.77 (17)
C14—C9—C10	117.4 (2)	C5—N2—C4	117.6 (2)
C14—C9—C15	122.8 (3)	C5—N2—Ru1	113.90 (17)
C10—C9—C15	119.8 (2)	C4—N2—Ru1	128.38 (16)
C14—C9—Ru1	70.90 (14)	F6—P1—F4	90.27 (19)
C10—C9—Ru1	70.05 (14)	F6—P1—F3	89.3 (2)
C15—C9—Ru1	130.05 (19)	F4—P1—F3	178.93 (15)
C11—C10—C9	121.8 (3)	F6—P1—F5	179.13 (19)
C11—C10—Ru1	72.07 (14)	F4—P1—F5	88.87 (16)
C9—C10—Ru1	72.03 (14)	F3—P1—F5	91.57 (18)
C11—C10—H10	119.1	F6—P1—F2	90.33 (16)
C9—C10—H10	119.1	F4—P1—F2	90.70 (13)
Ru1—C10—H10	129.3	F3—P1—F2	88.33 (13)
C10—C11—C12	120.4 (3)	F5—P1—F2	89.85 (14)
C10—C11—Ru1	70.78 (15)	F6—P1—F1	90.38 (15)
C12—C11—Ru1	71.39 (15)	F4—P1—F1	90.59 (13)
C10—C11—H11	119.8	F3—P1—F1	90.39 (13)
C12—C11—H11	119.8	F5—P1—F1	89.46 (13)
Ru1—C11—H11	130.7	F2—P1—F1	178.53 (13)
C13—C12—C11	118.1 (2)	N1—Ru1—N2	77.91 (8)
C13—C12—C18	121.5 (3)	N1—Ru1—C13	95.62 (9)
C11—C12—C18	120.3 (3)	N2—Ru1—C13	117.75 (9)
C13—C12—Ru1	70.36 (15)	N1—Ru1—C10	157.21 (10)
C11—C12—Ru1	70.77 (15)	N2—Ru1—C10	124.11 (10)
C18—C12—Ru1	128.85 (18)	C13—Ru1—C10	79.68 (10)
C12—C13—C14	121.1 (3)	N1—Ru1—C14	95.53 (9)
C12—C13—Ru1	72.49 (15)	N2—Ru1—C14	154.82 (9)
C14—C13—Ru1	71.58 (14)	C13—Ru1—C14	37.97 (10)
C12—C13—H13	119.4	C10—Ru1—C14	67.02 (10)
C14—C13—H13	119.4	N1—Ru1—C11	158.10 (9)
Ru1—C13—H13	128.9	N2—Ru1—C11	97.57 (9)
C9—C14—C13	121.2 (3)	C13—Ru1—C11	67.23 (10)
C9—C14—Ru1	71.98 (15)	C10—Ru1—C11	37.15 (10)
C13—C14—Ru1	70.45 (15)	C14—Ru1—C11	79.41 (10)
C9—C14—H14	119.4	N1—Ru1—C12	120.53 (9)
C13—C14—H14	119.4	N2—Ru1—C12	94.32 (9)
Ru1—C14—H14	130.9	C13—Ru1—C12	37.15 (11)
C9—C15—C17	114.1 (2)	C10—Ru1—C12	67.90 (10)
C9—C15—C16	108.1 (2)	C14—Ru1—C12	67.84 (10)
C17—C15—C16	112.0 (3)	C11—Ru1—C12	37.84 (10)
C9—C15—H15	107.5	N1—Ru1—C9	119.65 (9)
C17—C15—H15	107.5	N2—Ru1—C9	161.77 (10)
C16—C15—H15	107.5	C13—Ru1—C9	68.20 (10)
C15—C16—H16A	109.5	C10—Ru1—C9	37.92 (11)
C15—C16—H16B	109.5	C14—Ru1—C9	37.11 (10)
H16A—C16—H16B	109.5	C11—Ru1—C9	68.03 (10)
C15—C16—H16C	109.5	C12—Ru1—C9	81.09 (10)
H16A—C16—H16C	109.5	N1—Ru1—Cl1	86.45 (6)
H16B—C16—H16C	109.5	N2—Ru1—Cl1	84.46 (6)
C15—C17—H17A	109.5	C13—Ru1—Cl1	157.67 (7)
C15—C17—H17B	109.5	C10—Ru1—Cl1	89.80 (7)
H17A—C17—H17B	109.5	C14—Ru1—Cl1	119.71 (7)
C15—C17—H17C	109.5	C11—Ru1—Cl1	114.67 (8)
H17A—C17—H17C	109.5	C12—Ru1—Cl1	152.21 (8)
H17B—C17—H17C	109.5	C9—Ru1—Cl1	91.43 (7)
			
N1—C1—C2—C8	1.0 (4)	C11—C12—C13—Ru1	−54.0 (2)
C8^i^—C3—C4—N2	−0.3 (4)	C18—C12—C13—Ru1	124.2 (2)
N2—C5—C6—N1	−0.1 (3)	C10—C9—C14—C13	1.5 (4)
C7—C5—C6—N1	179.9 (2)	C15—C9—C14—C13	−178.5 (2)
N2—C5—C6—C7^i^	179.2 (2)	Ru1—C9—C14—C13	−52.6 (2)
C7—C5—C6—C7^i^	−0.8 (4)	C10—C9—C14—Ru1	54.1 (2)
N2—C5—C7—C6^i^	−179.2 (2)	C15—C9—C14—Ru1	−125.9 (2)
C6—C5—C7—C6^i^	0.8 (4)	C12—C13—C14—C9	−1.9 (4)
N2—C5—C7—C8^i^	1.2 (4)	Ru1—C13—C14—C9	53.3 (2)
C6—C5—C7—C8^i^	−178.8 (2)	C12—C13—C14—Ru1	−55.2 (2)
C1—C2—C8—C7^i^	−1.0 (4)	C14—C9—C15—C17	37.2 (4)
C1—C2—C8—C3^i^	179.1 (2)	C10—C9—C15—C17	−142.9 (3)
C14—C9—C10—C11	−0.1 (4)	Ru1—C9—C15—C17	−54.7 (4)
C15—C9—C10—C11	180.0 (2)	C14—C9—C15—C16	−88.1 (3)
Ru1—C9—C10—C11	54.5 (2)	C10—C9—C15—C16	91.9 (3)
C14—C9—C10—Ru1	−54.5 (2)	Ru1—C9—C15—C16	−180.0 (3)
C15—C9—C10—Ru1	125.5 (2)	C7^i^—C6—N1—C1	−0.5 (4)
C9—C10—C11—C12	−1.1 (4)	C5—C6—N1—C1	178.8 (2)
Ru1—C10—C11—C12	53.4 (2)	C7^i^—C6—N1—Ru1	178.40 (18)
C9—C10—C11—Ru1	−54.4 (2)	C5—C6—N1—Ru1	−2.4 (3)
C10—C11—C12—C13	0.8 (4)	C2—C1—N1—C6	−0.2 (4)
Ru1—C11—C12—C13	53.8 (2)	C2—C1—N1—Ru1	−178.90 (19)
C10—C11—C12—C18	−177.5 (2)	C7—C5—N2—C4	−1.2 (4)
Ru1—C11—C12—C18	−124.4 (2)	C6—C5—N2—C4	178.8 (2)
C10—C11—C12—Ru1	−53.1 (2)	C7—C5—N2—Ru1	−177.53 (18)
C11—C12—C13—C14	0.7 (4)	C6—C5—N2—Ru1	2.5 (3)
C18—C12—C13—C14	179.0 (2)	C3—C4—N2—C5	0.8 (4)
Ru1—C12—C13—C14	54.7 (2)	C3—C4—N2—Ru1	176.49 (19)

Symmetry code: (i) −*x*+1, −*y*+1, −*z*.

### Hydrogen-bond geometry (Å, º)

**Table:**

*D*—H···*A*	*D*—H	H···*A*	*D*···*A*	*D*—H···*A*
C1—H1···O1	0.93	2.39	3.194 (4)	145
C3—H3···Cl1^ii^	0.93	2.84	3.637 (3)	145
C3—H3···F6^iii^	0.93	2.58	3.205 (4)	125
C4—H4···F6^iii^	0.93	2.60	3.223 (4)	125
C19—H19*B*···F4^iv^	0.96	2.46	3.321 (5)	149

Symmetry codes: (ii) −*x*, −*y*+1, −*z*; (iii) *x*−1, *y*, *z*; (iv) *x*, *y*+1, *z*.
